# Outcomes of phacoemulsification and endoscopic cyclophotocoagulation performed with dual blade ab interno trabeculectomy or trabecular micro-bypass stent insertion

**DOI:** 10.1038/s41433-021-01475-4

**Published:** 2021-03-10

**Authors:** Emma Klug, Marika Chachanidze, Abraham Nirappel, Enchi K. Chang, Nathan Hall, Ta C. Chang, David Solá-Del Valle

**Affiliations:** 1 Massachusetts Eye and Ear, 243 Charles St, Boston, MA 02114 USA; 2 Bascom Palmer Eye Institute, 900 NW 17th St, Miami, FL 33136 USA

**Keywords:** Outcomes research, Surgery

## Abstract

**Background/Objective:**

To report the initial outcomes of phacoemulsification, endoscopic cyclophotocoagulation, and dual blade ab interno trabeculectomy (PEcK), and compare them to those of phacoemulsification, endoscopic cyclophotocoagulation, and trabecular micro-bypass stent insertion (ICE-1).

**Subjects/Methods:**

Patients from January 2018 to December 2019 that underwent PEcK or ICE-1 at a tertiary referral centre were included in this retrospective comparative case series. Patients were excluded if they had additional concomitant procedures, less than 6 weeks (42 days) of follow-up or were not at least 18 years old. Intraocular pressure (IOP), number of glaucoma medications, and best-corrected visual acuity were collected preoperatively and postoperatively at 6 weeks, 3, 6, and 12 months. Kaplan–Meier survival analysis and Cox proportional-hazards regression were conducted to elucidate any factors associated with survival time.

**Results:**

The mean preoperative IOP was 18.3 ± 5.9 mmHg in the PEcK group (53 eyes) and 14.7 ± 4.3 mmHg in the ICE-1 group (23 eyes) (*p* = 0.004) on 3.3 ± 1.3 and 1.7 ± 0.93 glaucoma medications (*p* < 0.001), respectively. Twelve months postoperatively the mean IOP reduction was 5.1 ± 4.4 mmHg and 2.3 ± 4.0 mmHg (*p* = 0.08), and the mean medication reduction was 1.6 ± 1.5 and 0.97 ± 0.66 (*p* = 0.10), in the PEcK and ICE-1 groups, respectively. Kaplan–Meier survival analysis did not reveal any differences in treatment survival.

**Conclusions:**

Both PEcK and ICE-1 provide clinically relevant reductions in IOP and glaucoma medication burden, however the PEcK procedure may confer greater reductions in IOP. The procedures did not differ with regard to Kaplan–Meier survival probability.

## Introduction

In recent years, microinvasive glaucoma surgery (MIGS) has emerged as a potentially favorable alternative to traditional glaucoma filtration surgery for patients with mild or moderate glaucoma [1]. For these patients, MIGS can offer reductions in both intraocular pressure (IOP) and glaucoma medication burden as well as an excellent safety profile [1]. Moreover, there is a unique opportunity to combine multiple MIGS that lower IOP via different mechanisms of action. Specifically, the glaucoma surgeon may combine procedures that simultaneously decrease aqueous production and increase its outflow, potentially leading to additive reductions in IOP and dependence on glaucoma medications.

The generation 1, single iStent® device (Glaukos Corp.) has been combined with endoscopic cyclophotocoagulation (ECP) and phacoemulsification cataract surgery in what is known as the ICE-1 procedure [2]. The iStent is an ab interno micro-stent designed to serve as a bypass through the trabecular meshwork (TM) to improve aqueous outflow. In turn, ECP allows for controlled and targeted inactivation of the ciliary body through direct visualization and coagulation, thereby reducing aqueous production. Recently, Ferguson et al. demonstrated that combining these three modalities in the ICE-1 procedure provided significantly better IOP reduction compared to phacoemulsification and ECP alone [2].

The Kahook dual blade (KDB; New World Medical Inc.) is a novel trabeculectomy blade that increases aqueous humor outflow, and it can be combined with ECP and phacoemulsification in a similar manner. While this procedure has not yet been described in the literature, ECP and KDB have both been shown to effectively reduce IOP when performed individually with concomitant phacoemulsification [3, 4].

To the best of our knowledge, there is no current literature reporting the outcomes of combined Phacoemulsification, ECP, and KDB ab interno trabeculectomy, herein termed the *PEcK* procedure. As ICE-1 has been shown to confer some additional efficacy compared to phacoemulsification and stent insertion alone, it follows that PEcK may have similar efficacy without the risks of malposition or malfunction that can be associated with trabecular implants. Therefore, the purpose of the current retrospective study is twofold; first, we report the initial outcomes of the PEcK procedure, and second, we compare them to those of the ICE-1 procedure.

## Methods

### Study design

Approval for this retrospective review was obtained from the Mass General Brigham Institutional Review Board. All research adhered to the tenets of the Declaration of Helsinki and was compliant with the Health Insurance Portability and Accountability Act. Consecutive patients who underwent PEcK or ICE-1 were identified by review of a single surgeon’s operating history between January 2018 and December 2019. Patients included in the analysis underwent PEcK or ICE-1 to reduce IOP or topical glaucoma medication burden. Patients were excluded from the analysis if they had less than 6 weeks (42 days) of follow-up at our institution, had any additional procedures at the time of PEcK or ICE-1, or if they were not at least 18 years of age at the time of surgery. If both eyes were treated with the same combination of procedures, only the right eye was included in the analysis. If patients had discordant procedures between the two eyes (i.e., PEcK in one eye, ICE-1 in the other) the first eye to undergo surgery was included in its respective group, and the fellow eye was excluded.

Preoperative data collected included patient age, gender, glaucoma diagnosis, number of glaucoma medications (number of constituent agents if fixed-dose combination medications were used), best-corrected visual acuity (BCVA), and IOP. IOP was measured by the surgeon using Goldmann applanation tonometry. The average of the measurements taken on two consecutive visits prior to surgery was used for the baseline IOP. Glaucoma stage was assigned in accordance with the recommendations put forth in the American Academy of Ophthalmology Preferred Practice Pattern guidelines (ICD-10 Glaucoma Reference Guide). Patients were assigned mild-to-moderate or moderate-to-severe glaucoma when severity fluctuated as a result of fluctuating optical coherence tomography and Humphrey visual field findings. Postoperative data collected included IOP, BCVA, number of glaucoma medications, duration of follow-up, presence of any postoperative complications related to the study procedures, and any subsequent surgical interventions required to manage complications or control IOP. Postoperative data were collected from follow-up evaluations at 6 (±2.5) weeks, 3 (±1), 6 (±2), and 12 (±3) months.

### Main outcome measures

The main outcome measures were Kaplan–Meier survival probabilities with failure of treatment defined in two ways:

Criteria 1—continued uncontrolled IOP >21 mmHg, or IOP reduction <20% from preoperative baseline, or the addition of glaucoma medications from baseline on two consecutive follow-up visits after 30 days OR need for additional glaucoma surgery and;

Criteria 2—failure to reach a preoperatively designated goal IOP, or if patients were at goal IOP preoperatively on glaucoma medications, failure to maintain goal IOP while reducing glaucoma medication burden on two consecutive follow-up visits after 30 days OR need for additional glaucoma surgery.

The preoperatively designated goal IOP was defined by the surgeon prior to the surgery, and it corresponded to a 20% IOP reduction from the level at which glaucoma progression was first documented.

### Surgical technique

#### PEcK procedure

All procedures were performed by a single attending (DSD) in the Glaucoma Service at Massachusetts Eye and Ear. Following phacoemulsification, the ECP probe was inserted into the sulcus and 120–360° of ciliary processes were treated in continuous-wave mode. ECP power was titrated between 0.25 and 0.50 W until whitening and shrinkage of the ciliary processes were observed. After rotating the patient’s head for visualization of the angle, the gonioscopy lens was placed onto the cornea with viscoelastic material in the interface. When excellent visualization was achieved, the KDB was introduced into the anterior chamber and an ab interno trabeculectomy was performed. The KDB was passed through the TM between 3.5 and 5.0 clock hours in an inside-out fashion until two strips of TM were formed. Intracameral antibiotics and Miochol were injected into the anterior chamber (0.1 cc moxifloxacin or cefuroxime, 0.2 cc Miochol). A subconjunctival injection of dexamethasone, and a drop of prednisolone and antibiotic were placed on the eye before it was patched and shielded.

#### ICE-1 procedure

Following phacoemulsification, the ECP probe was inserted into the sulcus and 180–270° of ciliary processes were treated in continuous-wave mode. ECP power was titrated between 0.25 and 0.50 W until whitening and shrinkage of the ciliary processes were observed. After rotating the patient’s head to visualize the angle, the gonioscopy lens was placed onto the cornea with viscoelastic material in the interface. When excellent visualization was achieved, the generation 1 iStent was inserted into the nasal TM, and the proper check procedures were performed to confirm secure placement. Intracameral antibiotics and Miochol were injected into the anterior chamber (0.1 cc moxifloxacin or cefuroxime, 0.2 cc Miochol). A subconjunctival injection of dexamethasone, and a drop of prednisolone and antibiotic were placed on the eye before it was patched and shielded.

### Data analysis

For preoperative and postoperative comparisons, the paired Student’s *t-*test was used for IOP, number of medications, and BCVA at 6 weeks, 3, 6, and 12 months. Snellen BCVA was converted to logarithm of the minimum angle of resolution (logMAR) for the analysis. For comparisons between groups, Student’s *t*-test was used for age, IOP, number of medications, and logMAR visual acuity. The Chi-squared test and the Fisher exact test were used for frequencies or proportions (gender, glaucoma diagnosis, stage, and complications). If a patient required a subsequent IOP-lowering procedure, they were excluded from any analyses following the date of the second procedure. Kaplan–Meier survival curves were created for both procedures based on two different failure criteria, and log-rank tests were used to compare the survival functions. Both univariate and multivariate Cox proportional-hazards regression models were used to elucidate any factors associated with failure. Statistical significance was defined as *P* < 0.05. Statistical analysis was performed with R statistical programming (R version 4.0.0, 2020-04-24).

## Results

Overall, 76 eyes of 76 patients were included in the study; 53 underwent PEcK and 23 underwent ICE-1. Twenty-one percent (11/53) of PEcK patients and thirty-four percent (8/23) of ICE-1 patients underwent concordant bilateral procedures, and the right eye was chosen for analysis. Two of seventy-six patients (2.6%) underwent both PEcK and ICE-1. The first eye to undergo surgery was included in its respective group, and the fellow eye was excluded. Patient demographics are given in Table 1. There were no significant differences in age, sex, or race between the two groups. Follow-up ranged from 43 to 702 days in the PEcK group with a mean follow-up of 226 (±161) days. Follow-up ranged from 50 to 685 days in the ICE-1 group with a mean follow-up of 305 (±152) days. In the PEcK group, a mean of 203 (±39) degrees of ciliary processes was treated with a mean power of 0.35 (0.05) Watts. A mean of 4 clock hours of TM were excised. In the ICE-1 group, a mean of 195 (±21) degrees of ciliary processes were treated with a mean power of 0.34 (0.03) Watts of laser power.

**Table 1:** Baseline demographics and glaucoma type and stage.

	PEcK	ICE-1	*p* value^*^
*N*	53	23	
Age (years; mean ± SD)	72.2 (7.3)	72.8 (8.3)	0.73
Gender, *n* (%)			
Female	24 (45.3)	15 (65.2)	
Male	29 (54.7)	8 (34.8)	0.12
Race, %			
Caucasian	71.7	60.9	0.10
Black or African American	13.2	8.7	
Hispanic	11.3	21.7	
Asian	0	8.7	
Unknown	3.8	0	
Glaucoma type, %			0.02^*^
Primary open angle	50.9	30.4	
Pigmentary	3.7	0	
Normal tension	5.7	21.7	
Secondary open angle	0	13.1	
Traumatic	0	4.3	
Pseudoexfoliation	17	8.7	
Mixed mechanism	17	13.1	
Ocular hypertension	5.7	8.7	
Glaucoma stage, %			<0.001^*^
Mild	17	30.4	
Mild-to-moderate	11.3	47.8	
Moderate	26.4	13	
Moderate-to-severe	30.2	0	
Severe	9.4	0	

Baseline demographics and proportions of glaucoma type and stage in the phacoemulsification, endoscopic cyclophotocoagulation, and dual blade ab interno trabeculectomy (PEcK) and the phacoemulsification, endoscopic cyclophotocoagulation, and trabecular micro-bypass stent (ICE-1) groups.

^*^Statistical significance.

### Baseline characteristics

While the groups had similar BCVA at baseline, the PEcK group had significantly higher IOP (*p* = 0.004; Table 2) and was on more glaucoma medications (*p* < 0.001; Table 3). The PEcK group also consisted of a smaller proportion of patients with mild glaucoma compared to the ICE-1 group (*p* < 0.001; Table 1). The distribution of glaucoma type also differed between groups, with more cases of primary open-angle glaucoma in the PEcK group (*p* = 0.02; Table 1).

### IOP, glaucoma medications, and BCVA

Postoperative IOP was significantly reduced from baseline at all time points in the PEcK group (*p* < 0.001 at all time points), and at postoperative week 6 in the ICE-1 group (*p* = 0.04; Table 2). The mean reduction in IOP was significantly greater in the PEcK group compared to the ICE-1 group at postoperative week 6 (*p* = 0.01), month 3 (*p* = 0.005), and month 6 (*p* = 0.002).

**Table 2:** Intraocular pressure.

IOP (mmHg)	PEcK				ICE-1				*p* value (between groups)*
	*N*	Mean (±SD)	Mean reduction from baseline (±SD)	*p* value (from baseline)*	*N*	Mean (±SD)	Mean reduction from baseline (±SD)	*p* value (from baseline)*	
Baseline	53	18.3 (5.9)			23	14.7 (4.3)			0.004*
6 weeks	48	13.5 (3.9)	4.2 (4.1)	<0.001*	21	12.9 (2.7)	1.7 (3.5)	0.04*	0.01*
3 months	21	13.3 (2.9)	6.3 (6.4)	<0.001*	15	12.4 (2.1)	1.3 (3.7)	0.2	0.005*
6 months	35	13.2 (2.9)	5.0 (4.9)	<0.001*	19	14.1 (4.2)	0.67 (4.5)	0.52	0.002*
12 months	17	14.1 (3.4)	5.1 (4.4)	<0.001*	12	13.3 (2.8)	2.3 (4.0)	0.08	0.08

Mean intraocular pressure (IOP) and mean reduction in IOP at postoperative week 6, months 3, 6, and 12 in the phacoemulsification, endoscopic cyclophotocoagulation, and dual blade ab interno trabeculectomy (PEcK) and the phacoemulsification, endoscopic cyclophotocoagulation, and trabecular micro-bypass stent (ICE-1) groups. Significance was calculated from baseline for each procedure individually, and between the relative reductions in IOP at each time point for the two procedures.

^*^Statistical significance.

Postoperative glaucoma medication burden was significantly reduced from baseline in the PEcK and ICE-1 groups independently at all time points (PEcK and ICE-1, *p* < 0.001 at all time points; Table 3). The mean reduction in medications was greater in the PEcK group compared to the ICE-1 group, however this difference only approached statistical significance at postoperative week 6 (*p* = 0.08) and postoperative month 6 (*p* = 0.07).

**Table 3:** Glaucoma medications.

Glaucoma medications	PEcK				ICE-1				*p* value (between groups)^*^
	*N*	Mean (±SD)	Mean reduction from baseline (±SD)	*p* value (from baseline)^*^	*N*	Mean (±SD)	Mean reduction from baseline (±SD)	*p* value (from baseline)^*^	
Baseline	53	3.3 (1.3)			23	1.7 (0.93)			<0.001^*^
6 weeks	48	1.6 (1.3)	1.7 (1.3)	<0.001^*^	21	0.52 (0.75)	1.2 (0.76)	<0.001^*^	0.08
3 months	21	1.3 (1.1)	1.8 (1.2)	<0.001^*^	15	0.40 (0.51)	1.3 (0.61)	<0.001^*^	0.11
6 months	35	1.7 (1.2)	1.7 (1.2)	<0.001^*^	19	0.54 (0.75)	1.3 (0.52)	<0.001^*^	0.07
12 months	17	1.7 (1.2)	1.6 (1.5)	<0.001^*^	12	0.94 (0.94)	0.97 (0.66)	<0.001^*^	0.10

Mean number of glaucoma medications and mean reduction in reduction in glaucoma medications at postoperative week 6, months 3, 6, and 12 in the phacoemulsification, endoscopic cyclophotocoagulation, and dual blade ab interno trabeculectomy (PEcK) and the phacoemulsification, endoscopic cyclophotocoagulation, and trabecular micro-bypass stent (ICE-1) groups. Significance was calculated from baseline for each procedure individually, and between the relative reductions in glaucoma medications at each time point for the two procedures.

^*^Statistical significance.

BCVA was significantly improved from baseline at postoperative week 6 (*p* = 0.01) and postoperative month 6 (*p* = 0.002) in the PEcK group, and at postoperative week 6 (*p* = 0.01), month 3 (*p* = 0.005), and month 6 (*p* = 0.01) in the ICE-1 group. There were no between-group differences in the mean change in BCVA at any time point.

### Survival analysis—failure criteria 1

**Fig. 1:**
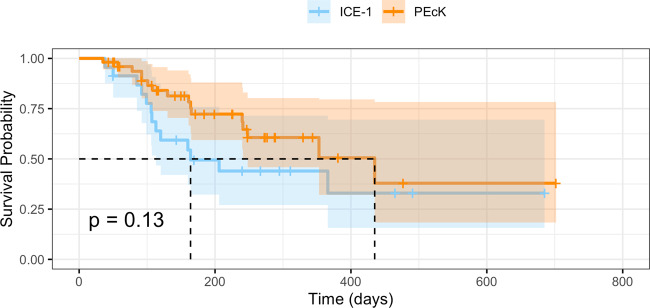
Kaplan–Meier survival analysis corresponding to failure criteria 1 for the phacoemulsification, endoscopic cyclophotocoagulation, and dual blade ab interno trabeculectomy (PEcK) and the phacoemulsification, endoscopic cyclophotocoagulation, and trabecular micro-bypass stent (ICE-1) groups. There is no statistically significant difference between the survival functions (log-rank test, *p* = 0.12).

The Kaplan–Meier survival functions corresponding to failure criteria 1 are shown in Fig. 1. The median survival time was 453 days in the PEcK group and 164 days in the ICE-1 group. The cumulative probability of success in the PEcK group was 72% (95% confidence interval [CI] 0.59–0.88) at 6 months, and 51% (CI 0.32–0.80) at 12 months postoperatively. The cumulative probability of success in the ICE-1 group was 50% (CI 0.32–0.76) at 6 months, and 44% (CI 0.27–0.72) at 12 months. There was no significant difference between the survival functions of the PEcK and ICE-1 groups (*p* = 0.13, log-rank test).

Univariate Cox proportional-hazard analyses were conducted to elucidate whether individual factors were associated with failure for both procedures. Hispanic race was significantly associated with treatment failure in both groups. Hispanic race demonstrated a hazard ratio (HR) of 6.23 (CI 1.59–25.0) (*p* = 0.009) and 4.56 (CI 1.33–15.68) (*p* = 0.02) in the PEcK and ICE-1 groups, respectively. Higher baseline IOP demonstrated a lower hazard of failure (HR of 0.72 [CI 0.59–0.89]) (*p* = 0.002) in the ICE-1 group, however no significant effect was observed in the PEcK group (HR 0.93 [CI 0.84–1.0]) (*p* = 0.16). Neither sex, age, glaucoma stage nor baseline glaucoma medications were significantly associated with failure for either procedure.

A multivariate Cox proportional-hazards model for failure criteria 1 was created using procedure type, baseline IOP, baseline number of medications, and glaucoma stage as covariates. Higher baseline IOP demonstrated a lower hazard of failure (HR 0.87 [CI 0.79–0.96]) (*p* = 0.005). All other covariates were nonsignificant.

### Survival analysis—failure criteria 2

**Fig. 2:**
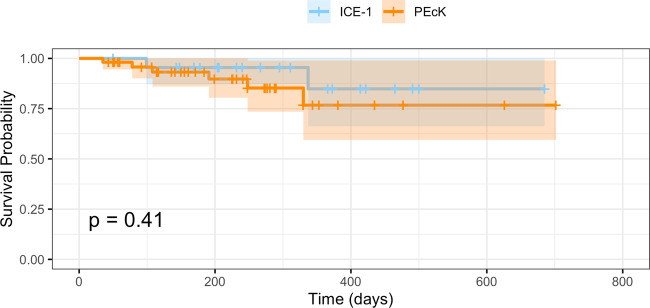
Kaplan–Meier survival analysis corresponding to failure criteria 2 for the phacoemulsification, endoscopic cyclophotocoagulation, and dual blade ab interno trabeculectomy (PEcK) and the phacoemulsification, endoscopic cyclophotocoagulation, and trabecular micro-bypass stent (ICE-1) groups. There is no statistically significant difference between the survival functions (log-rank test, *p* = 0.41).

The Kaplan–Meier survival functions for failure criteria 2 are shown in Fig. 2. The cumulative probability of success in the PEcK group was 93% (CI 0.86–1.0) at 6 months, and 77% (CI 0.59–0.99) at 12 months postoperatively. The cumulative probability of success in the ICE-1 group was 96% (CI 0.87–1.0) at 6 months, and 85% (CI 0.66–1.0) at 12 months. There was not a significant difference in the survival functions of the two procedures based on failure criteria 2 (*p* = 0.41, log-rank test).

Univariate Cox proportional-hazards analyses were also conducted in accordance with failure criteria 2. Neither baseline IOP, baseline glaucoma medications, sex, age, race nor glaucoma stage were significantly associated with failure for either procedure independently. A multivariate Cox proportional-hazards model for failure criteria 2 was created using procedure type, baseline IOP, baseline number of medications, and glaucoma stage as covariates. No covariates were found to have a significant effect on survival.

### Postoperative complications

In both groups, incidences of mild postoperative hyphema, inflammation, and corneal edema were rare, and all resolved spontaneously by month 3. Eight percent (4/53) of patients in the PEcK group required a subsequent IOP-lowering procedure (one Ahmed glaucoma valve, two Baerveldt glaucoma implants, one trabeculectomy). Six percent (3/53) of PEcK patients developed a visually significant posterior capsular opacification that required an Nd:YAG capsulotomy. There were no statistically significant differences between groups with regard to any postoperative complications or the need for additional procedures.

## Discussion

To the best of our knowledge, the PEcK procedure has not yet been described in the literature. In the current study, the PEcK procedure resulted in significant reductions from baseline in both IOP and glaucoma medication burden at all postoperative time points. There is evidence that phacoemulsification alone can provide modest reductions in IOP. Specifically, 12 months after phacoemulsification, Majstruk et al. found that IOP was reduced by 1.15 mmHg, while medication burden remained unchanged [5]. Further, combining phacoemulsification with ECP (phaco-ECP) or KDB (phaco-KDB) have both been shown to confer greater reductions in IOP and medication burden than phacoemulsification alone. Studies of phaco-ECP have reported IOP reductions ranging from 2.7 to 4.7 mmHg, and medication reductions ranging from 0.4 to 0.9 [4, 6, 7]. Similar reductions have been reported in studies of phaco-KDB, with reductions in IOP ranging from 2.1 to 4.4 mmHg, and reductions in medications ranging from 0.4 to 1.2 [3, 8–11]. In the current study, the PEcK group demonstrated a slightly greater reduction in IOP and a substantially greater reduction in glaucoma medication burden at 12 months postoperatively compared to previous studies of phacoemulsification alone, phaco-ECP, and phaco-KDB.

Moreover, compared to the ICE-1 group, PEcK demonstrated significantly greater reductions in IOP up to 6 months postoperatively. This difference approached statistical significance at postoperative month 12. This could potentially be attributable to the larger opening in the TM created by ab interno trabeculectomy with the KDB compared to the iStent. However, the PEcK group also had significantly greater IOP at baseline compared to the ICE-1 group. As such, the observed differences in IOP reduction must be interpreted with caution.

In addition, as is expected following cataract surgery, BCVA was significantly improved from baseline in the PEcK group at postoperative week 6 and month 6, and it was improved in the ICE-1 group at postoperative week 6, month 3 and month 6. Visual acuity improvement likely did not reach statistical significance at postoperative month 3 or 12 in the PEcK group, or month 12 in the ICE-1 group, due to small sample sizes.

As previously mentioned, only one other study has reported the outcomes of the ICE-1 procedure. In their study of ICE-1 versus phacoemulsification and iStent (phaco-iStent) alone, Ferguson et al. reported a mean 7.13 mmHg reduction in IOP and a 0.68 reduction in glaucoma medications 12 months after ICE-1. This reduction in IOP is substantially greater than that of the current ICE-1 group at 12 months, however we did observe a slightly greater reduction in medication burden. The difference in IOP reduction may be attributable to differences in patient populations. Specifically, Ferguson et al. reported a preoperative IOP of 21 mmHg and only included patients with open-angle glaucoma. In contrast, the baseline IOP of the current ICE-1 group was just 14.7 mmHg, and no exclusions were made based on the type of glaucoma. It has been previously demonstrated that higher initial IOP will result in greater posttreatment reduction [3, 12].

In addition, there is evidence that 360° of ECP treatment is superior to 180° with regard to IOP reduction [13]. In the current study, a mean of 203° of ciliary processes were treated in the ICE-1 group, while Ferguson et al. treated 270° of ciliary processes [2]. This discrepancy could have also contributed to the greater reduction in IOP observed by Ferguson et al. with greater reductions observed after larger numbers of ciliary processes are treated.

Two Kaplan–Meier survival curves were created for both procedures to determine the cumulative probability of treatment success over time. Based on failure criteria 1, the median survival time was greater in the PEcK group compared to the ICE-1 group, however the log-rank test did not detect a statistical difference between the two survival functions. Univariate Cox proportional-hazards analyses were conducted to elucidate any factors that may be associated with the success of each procedure individually. Hispanic race was strongly associated with increased risk of failure based on failure criteria 1, increasing the risk of failure by 523% in the PEcK group and 356% in the ICE-1 group.

There is currently little to no literature relating race to glaucoma surgery outcomes. However, it is well-established that there is an increased prevalence of glaucoma amongst Hispanic populations compared to other racial groups, and they have been shown to present with more severe disease [14]. Studies have shown that Hispanic individuals have the lowest rates of visual field testing compared to White, Black, or Asian individuals, and are less likely to receive medical or surgical intervention [15, 16]. Our findings may suggest that disparities in glaucoma treatment in Hispanic populations affect their surgical outcomes.

To account for differences in baseline characteristics between the two groups, a multivariate Cox proportional-hazards regression was used to simultaneously relate the procedure type, baseline IOP, baseline number of medications, and glaucoma stage to failure. Due to a wide range of glaucoma diagnoses and modest sample sizes, our study was not sufficiently powered to account for baseline differences in the distribution of glaucoma type. Only baseline IOP was significantly associated with survival. Specifically, higher baseline IOP reduced the risk of treatment failure by 13%. As previously mentioned, higher baseline IOP is associated with greater posttreatment reductions in IOP. This may suggest that patients with higher baseline IOP were more likely to achieve a ≥20% reduction. Importantly, when the model was adjusted for all these factors simultaneously, it did not reveal any association between treatment type (PEcK or ICE-1) and survival time. This reinforces the nonsignificant finding between the survival functions of the two procedures, and it does not suggest one procedure is favorable to the other based on these survival criteria.

As the majority of patients in both groups had mild or moderate disease, the same failure criteria used in studies of traditional glaucoma filtration surgery (IOP > 21 mmHg or not reduced by 20%, Tube versus Trabeculectomy Study) may not be applicable [17]. Therefore, a second survival analysis was conducted using failure criteria 2. In this analysis, both procedures demonstrated excellent survival profiles, with survival probability remaining at or above 77% throughout follow-up. As with failure criteria 1, there was no difference between the survival functions for the two procedures, and the multivariate Cox proportional-hazards model did not reveal a relationship between procedure type and treatment survival.

With regard to postoperative complications, both the PEcK and ICE-1 procedures exhibited low rates of postoperative inflammation, hyphema, and corneal oedema that all subsided without intervention by postoperative month 3. While the rates of PCO that required an Nd:YAG capsulotomy did not differ significantly between the groups, the 6% rate observed in the PEcK group is slightly higher than that previously reported following phacoemulsification alone. Specifically, in a study of 55,567 cataract surgeries, 1.22% developed PCO that indicated a capsulolotomy [18]. More research is needed to assess whether combining multiple MIGS with phacoemulsification may increase the risk of developing a visually significant PCO.

The PEcK procedure may offer advantages compared to the ICE-1 procedure. Specifically, while the PEcK group was comprised of more patients with moderate and moderate-to-severe glaucoma, PEcK outperformed ICE-1 with regard to mean IOP reduction up to 6 months postoperatively. PEcK also appears to confer greater reductions in both IOP and medication burden compared to previous studies of phacoemulsification, phaco-ECP, and phaco-KDB. In addition, the PEcK procedure does not require the permanent implantation of a trabecular device. This particular safety advantage avoids the risk of device malposition or malfunction. When considered together, these factors may weigh in favor of the PEcK procedure as opposed to the ICE-1 procedure.

This study has several notable limitations, many of which can be attributed to its retrospective, non-randomized design. Specifically, the sample size is modest for both groups. Due to its retrospective nature, it relied on patient follow-up for data at each time point. The samples used for the comparisons of means experienced substantial attrition as we approached the 12-month mark. With such small sample sizes, caution must be taken in generalizing these results to the population at large.

In addition, the lack of randomization of patients to treatment groups may introduce potential bias inherent to this study design. The decision to undergo either PEcK or ICE-1 was made jointly by the surgeon and the patient on a case-by-case basis, while considering factors, such as IOP, medication burden, and patient preference with regard to recovery time and postoperative care. To avoid bias where possible, the first eye to undergo surgery was included in this analysis if a patient underwent PEcK and ICE-1 in different eyes, perhaps due to an inadequate response to treatment in the first eye. Moreover, baseline demographics, IOP, and glaucoma medications were unbalanced between groups. However, the multivariate Cox proportional-hazards model adjusted for these differences did not reveal an association between any these factors and the probability of survival.

There was no medication washout period at baseline or throughout follow-up. Due to the retrospective nature of this study, medication washout was neither a priority nor a safe option for patients. Lastly, IOP measurements were taken by the surgeon only at baseline and all postoperative time points, who inevitably was not masked to the surgical intervention.

Importantly, use of the first-generation iStent has been overwhelmingly replaced with the iStent *inject*® or iStent *inject*® W. These newer models were launched by Glaukos in the United States in 2018 and 2020, respectively. Both devices include two micro-bypass stents as opposed to one. The two-stent design of the iStent *injec*t has been shown to be more efficacious compared to the iStent with regard to reductions in IOP and glaucoma medication burden [19, 20]. At the time of this study, the surgeon had not performed enough ICE procedures using the iStent *inject* or iStent *inject* W to adequately power a study. However, the authors are actively pursuing a comparison of these newer models to the PEcK procedure.

In summary, in this retrospective study both PEcK and ICE-1 achieved clinically relevant reductions in IOP and glaucoma medication burden, and both procedures demonstrated favorable safety profiles. In addition, both procedures had favorable survival probability profiles, and differences in the survival functions were not apparent. The PEcK procedure may offer particular advantages compared to the ICE-1 procedure, as patients in this group had more severe glaucoma yet we observed noninferior reductions in IOP and glaucoma medication burden compared to ICE-1. In addition, the PEcK procedure does not involve the permanent placement of an ocular drainage device.

To determine if one of these combined MIGS procedures is truly superior to the other, a randomized controlled trial is necessary. However, to date randomized controlled trials investigating the efficacy of ECP or KDB alone are sorely lacking. These studies may take priority in order to fully elucidate any differences between the essential component-parts of these combined MIGS procedures.

### Summary

#### What was known before

- In glaucoma patients, combining phacoemulsification with Khaook dual blade ab interno trabeculectomy, iStent insertion, or endoscopic cyclophotocoagulation individually is effective in reducing intraocular pressure (IOP) and glaucoma medication burden. These combination procedures have been shown to reduce these measures more than phacoemulsification alone.
- Combining generation 1 iStent insertion, cataract extraction, and endoscopic cyclophotocoagulation in what is known as the ICE-1 procedure, has been shown to provide better IOP reductions compared to iStent insertion and phacoemulsification alone.
- Results of phacoemulsification, endoscopic cyclophotocoagulation, and Kahook dual blade ab interno trabeculectomy, herein termed the PEcK procedure, have not yet been reported in the literature. It is also unknown how this procedure compares to similar combined microinvasive surgical techniques, such as the ICE-1 procedure.

#### What this study adds

- Demonstrates for the first time that the PEcK procedure effectively reduces IOP and glaucoma medication burden and is relatively safe, with low rates of mild postoperative complications that resolved spontaneously.
- The PEcK procedure may offer particular benefits over the ICE-1 procedure, as it conferred greater reductions in IOP and does not involve the insertion of a permanent ocular device.
- Patients of Hispanic race undergoing PEcK or ICE-1 may require additional monitoring and consideration, as Hispanic race was significantly associated with an increased risk of treatment failure.
